# External Counterpulsation Improves Angiogenesis by Preserving Vascular Endothelial Growth Factor-A and Vascular Endothelial Growth Factor Receptor-2 but Not Regulating MicroRNA-92a Expression in Patients With Refractory Angina

**DOI:** 10.3389/fcvm.2021.761112

**Published:** 2021-10-25

**Authors:** Ade Meidian Ambari, Gracia Lilihata, Ervan Zuhri, Elok Ekawati, Shoma Adhi Wijaya, Bambang Dwiputra, Renan Sukmawan, Basuni Radi, Sofia Mubarika Haryana, Suko Adiarto, Dicky A. Hanafy, Dian Zamroni, Elen Elen, Arwin S. Mangkuanom, Anwar Santoso

**Affiliations:** ^1^Department of Cardiology and Vascular Medicine, Faculty of Medicine Universitas Indonesia - National Cardiovascular Center Harapan Kita, Jakarta, Indonesia; ^2^Division of Cardiovascular Research and Development, National Cardiovascular Center Harapan Kita, Jakarta, Indonesia; ^3^Department of Histology and Cell Biology, Faculty of Medicine, Public Health and Nursing, Universitas Gadjah Mada, Yogyakarta, Indonesia

**Keywords:** external counter pulsation (ECP), vascular endothelial growth factor (VEGF), vascular endothelial growth factor-A (VEGF-A), vascular endothelial growth factor receptor-2 (VEGFR-2), micro RNA-92a (miR-92a), angiogenesis

## Abstract

**Objective:** External counterpulsation (ECP) provides long-term benefits of improved anginal frequency and exercise tolerance in patients with refractory angina (RA). This is postulated as a result of improved angiogenesis and endothelial function through an increase in shear stress. Angiogenesis is mainly represented by vascular endothelial growth factor-A (VEGF-A) and its receptor, vascular endothelial growth factor receptor-2 (VEGFR-2). The microRNA-92a (miR-92a) is a flow-sensitive miRNA that regulates atherosclerosis and angiogenesis in response to shear stress. Thus, ECP beneficial effect might be achieved through interaction between VEGF-A, VEGFR-2, and miR-92a. This study aims to evaluate the ECP effect on VEGF-A, VEGFR-2, and miR-92a in patients with RA in a sham-controlled manner.

**Methods:** This was a randomized sham-controlled trial, enrolling 50 patients with RA who have coronary artery disease (CAD). Participants were randomized (1:1 ratio) to 35 sessions of either ECP (*n* = 25) or sham (*n* = 25), each session lasting for 1 h. Plasma levels of VEGF-A and VEGFR-2 were assayed by the ELISA technique. The quantitative reverse transcription-polymerase chain reaction (qRT-PCR) was performed to measure miR-92a circulating levels in plasma.

**Result:** External counterpulsation significantly preserved VEGF-A and VEGFR-2 level compared to sham [ΔVEGF-A: 1 (−139 to 160) vs.−136 (−237 to 67) pg/ml, *p* = 0.026; ΔVEGFR-2: −171(-844 to +1,166) vs. −517(−1,549 to +1,407) pg/ml, *p* = 0.021, respectively]. Circulating miR-92a increased significantly in ECP [5.1 (4.2–6.4) to 5.9 (4.8–6.4), p < 0.001] and sham [5.2 (4.1–9.4) to 5.6 (4.8–6.3), *p* = 0.008] post-intervention. The *fold changes* tended to be higher in ECP group, although was not statistically different from sham [*fold changes* ECP = 4.6 (0.3–36.5) vs. sham 2.8 (0–15), *p* = 0.33)].

**Conclusion:** External counterpulsation improved angiogenesis by preserving VEGF-A and VEGFR-2 levels. Both ECP and sham increased miR-92a significantly, yet the changes were not different between the two groups. (Study registered on www.clinicaltrials.gov, no: NCT03991871, August 8, 2019, and received a grant from the National Health Research and Development of Ministry of Health of Indonesia, No: HK.02.02/I/27/2020).

## Introduction

Refractory angina (RA), typically occurring in patients with advanced, often diffuse coronary artery disease (CAD), is described as angina that is still present despite optimal pharmacologic, intervention, or surgery which has been provided for >3 months (1). It is an emergent problem in patients with advanced CAD due to an aging population and improved survival from CAD (2, 3). Management of RA is aimed at toward reducing symptoms, improve quality of life (QoL), and preventing future cardiovascular events. Unfortunately, as many as 15% of the patients fail to respond to the management mentioned above or are ineligible to further intervention (2). Therefore, researchers are always trying to find new alternatives in clinical management.

External counterpulsation (ECP) is a noninvasive therapy employing external compressive cuffs on the calves and lower and upper thighs. It sequentially inflates cuffs from distal to proximal ends synchronized with the cardiac cycle detected by an electrocardiogram (ECG). The cuffs are inflated in early diastole to boost coronary artery perfusion, venous return, and then deflated simultaneously in systole to reduce systemic vascular resistance, cardiac workload, and enhance systemic perfusion (4). The effect of diastolic augmentation and increased coronary perfusion pressure was thought to be the reason for improvement in angina symptoms. However, improvement of symptoms was shown to persist for years after the completion of treatment, which could not be described by the acute hemodynamic effect only (5). Increased shear stress in coronary circulation by ECP treatment is then proposed as a principal underlying mechanism, resulting in multiple downstream favorable mechanisms, including an increase in angiogenesis, collateral circulation, improved endothelial function, and reduced arterial stiffness (4, 6, 7).

Improvement in angiogenesis has been indicated by many studies as one of the main mechanisms (8–12). Angiogenesis is mainly represented by vascular endothelial growth factor-A (VEGF-A) and vascular endovascular growth factor receptor-2 (VEGFR-2). There are several types of VEGF, including VEGF-A, VEGF-B, VEGF-C, VEGF-D, and VEGF-E. Among these types the VEGF-A plays the most important role in controlling angiogenesis (13). VEGF-A signaling goes through the class IV receptor tyrosine kinase group, the VEGF receptors (VEGFR). Although the VEGF-A ligand can bind to two VEGF-R receptors, namely VEGFR-1 and VEGFR-2, VEGF-A will primarily signal endothelial cell proliferation, survival, migration, and vascular permeability *via* VEGFR-2 (14). A previous study stated that ECP has a tendency for increasing VEGF (but not specifically VEGF-A) release in patients with CAD (15). However, there is no study that clearly and specifically evaluated the impact of ECP on VEGF-A and VEGFR-2 levels in a sham-controlled manner. Furthermore, high shear-stress flow has been shown by *in vitro* studies to decrease miRNA-92a (miR-92a), a pro-atherosclerotic and anti-angiogenesis miRNA (16). Due to scientific gaps in the effect of ECP on angiogenesis and miRNA-92a, this study aims to evaluate the effect of ECP therapy on angiogenesis represented by VEGF-A and VEGFR-2 and flow-sensitive miR-92a in patients with RA.

## Materials and Methods

### Study Design and Site of the Study

The HARTEC study was a randomized sham-controlled clinical trial with a parallel assignment to evaluate the effect of ECP therapy on angiogenesis, represented by the concentration of VEGF-A and VEGFR-2. The study also measured the expression of miR-92a since miR-92a is known as a mechano-miRNA that is affected by the high shear stress involved in the regulation of angiogenesis.

The study was conducted at the National Cardiovascular Center, Harapan Kita Hospital, Jakarta, Indonesia. This site was chosen because it is a tertiary hospital for cardiovascular care for most patients with severity levels-III (the highest level) referred from over the country. The study design has been registered on www.clinicaltrials.gov with an identifier number of NCT03991871 on August 8, 2019.

### Study Population

The study participants were patients with RA who did not respond to escalating medical treatment, determined by following the stepwise strategy of pharmacological agents based on the 2019 ESC Guidelines to diagnose and manage chronic coronary syndrome (17) after 3 months. Inclusion criteria were defined as the following: patients with angina pectoris aged 21–80 years old, suffering from RA with Canadian Cardiovascular Society (CCS) II–IV and who were not eligible for percutaneous coronary intervention (PCI) or coronary artery bypass grafting (CABG). The patients had stenosis on the left main (LM) coronary artery; more than 50% of patients had stenosis either on the main right coronary artery (RCA) and left anterior descending (LAD) artery, or more than 70% of them had left circumflex (LCX) artery. They were also not suitable candidates for further revascularization procedures decided in a presurgical conference in our hospital, or they refused any revascularization option. This conference was a regular meeting discussing the best option procedures in our hospital for patients with CAD.

Accordingly, the exclusion criteria were determined as follows: aorta and abdominal aneurysm, acute coronary syndrome within preceding 3 months, acute heart failure, severe aortic regurgitation, stage-III hypertension, peripheral artery disease, deep vein thrombosis, pregnancy, any surgical procedures in the preceding 6-weeks or cardiac catheterization in the preceding 2 weeks, bleeding diathesis, any fracture or burn wound hampered cuff compression, and finally arrhythmia unsynchronized with electrocardiogram (ECG). Our fellow clinicians screened for eligibility and only eligible patients were referred to our ECP clinic.

Basic demography, medical history, physical examination, medicinal use history, ECG, chest-x-ray, echocardiography, abdominal and vascular ultrasound, and Doppler study were conducted before randomization. Patients were to be reviewed for the WHO-5 Well-Being Index on QoL (18) and angina CCS class before and after the ECP and sham procedures. All the available data were stored in the HARTEC-database.

### Intervention and Sham-Procedures

A total of 50 patients were randomized and assigned to the ECP and sham groups. The randomization of generating the allocation sequence to the ECP or the sham group was carried out using computer-based random numbers. Furthermore, they were randomized in a 1:1 ratio to ECP or sham groups. The patients, outcome assessors, and data analysts were kept blinded to the assignment.

The patient randomized to the ECP group underwent a 35-hour of ECP sessions, consisting of 1 hour each day and 5-days per week from Monday to Friday, until 7 weeks. Three pairs of pneumatic cuffs were wrapped around the leg of the patient on calves, thighs, and buttocks while lying on a couch. The cuffs were inflated sequentially from distal to proximal ends at the onset of diastole. Then, they were deflated rapidly and simultaneously at the onset of systole. Protocol-specified applied pressure of up to 300 mmHg was provided within 5 min. The ECP device utilized was the Renew^TM^ NCP-5 (Singapore 554910, 2018). Patients assigned to the sham procedure underwent an identical treatment and frequency. The sham procedure was designed to be similar to ECP therapy, however with a pressure of only 75 mmHg to provide a feeling of being pressurized. Hence, patients would not realize which assignment they obtained.

The principal investigator did not know which patient was allocated. The procedures were operated on by another cardiologist registering in the area of cardiovascular disease (CVD) Prevention and Rehabilitation in our hospital. During the procedures, the vital signs and any adverse events were closely monitored and noted.

### Outcomes

Primary outcomes were the changes in VEGF-A, VEGFR-2, and miR-92a levels after completion of therapy compared to that in the baseline between ECP and sham groups. Secondary outcomes were CCS class changes, a 6-min walk test (6MWT), and WHO-5 Well Being Index improvement (18).

### Laboratory Examination

Patients were asked to come for blood sampling within 1 week before starting the treatment and 1 week after the completion of the treatment. Six milliliters of venous blood were drawn. The venous blood was kept in EDTA-tube; subsequently, plasma and peripheral blood mononuclear cells (PBMC) were separated by centrifuge within 30 min. Samples were centrifuged at 2,100 g for 10 min and then stored in multiple aliquots at −80°C and then assayed for VEGFR-2, VEGF-A, and miR-92a.

Vascular endothelial growth factor receptor-**2** analysis was performed on plasma samples using Human VEGFR2/KDR Quantikine ELISA Kit (Code: DVR 200, R&D Systems, Inc., MN, USA) according to the instructions of the manufacturer. The VEGF-A analysis was also carried out on the plasma sample using Human VEGF-A Elisa (Code: ELH-VEGF-1, RayBiotech, Inc, GA, USA) based on the instructions of the manufacturer. All the ELISA measurements were performed by individuals blinded to the clinical data of the patients.

According to the instructions of the manufacturer, total RNA was extracted using the miRNeasy Serum/Plasma kit (Qiagen, cat No 217184). The *cel-miR-*39 (Qiagen, cat. No 219610) was added as a control to correct for sample-to-sample variation (19). Then on 2 ng/ul of the sample, we performed reverse transcription using the TaqMan^TM^-MicroRNA Reverse Transcription Kit (ABI, Carslbard, CA, USA). Subsequently, a 2 μl complementary DNA (cDNA) sample (duplo) was used to detect miR-92a (ID 000431) with the corresponding TaqMan^TM^ microRNA assay kit by quantitative PCR (ABI 7500 Fast). The quantitative reverse transcription-polymerase chain reaction (qRT-PCR) was performed according to the recommendation of the manufacturer and in accordance with the previous study (19). The ΔCT of miR-92a was obtained after normalization to control as ΔCT = mean CT miR92a - mean CT *cel*-miR-39, and expressed as 2^−Δ*CT*.^ *Fold changes* of miR-92a after the procedure were expressed as 2^−Δ*ΔCT*^ (20, 21). Data were presented after transformation which was multiplied by 10^6^ and then log-transformed (22).

### Statistical Analysis

We performed an intention-to-treat analysis. All statistical analyses were performed using SPSS (version 25.0; SPSS Inc, Chicago, IL, 2017). Categorical variables were presented as numbers and percentages, while numerical variables were provided as mean and SD or median and minimum-maximum values if they were not normally distributed. Normality test was performed by Shapiro–Wilk test due to the small sample size.

A paired *t*-test compared the value of the change (before-after procedures), and the independent *t*-test was used to compare variables between the groups, if appropriate. Otherwise, a nonparametric test was used. Similarly, χ2 was used for binary and categorical variables. Multivariate analysis of covariance (MANCOVA) was performed applying the changes of VEGF-A, VEGFR-2, and miR-92a as dependent variables. The independent variable for the model was the ECP vs. sham procedures. If there is a variable imbalance between the groups, it should be constructed as a covariate model. The MANCOVA provides an overall test of significance, which gives an exact probability of the effect of the independent variable on the dependent variables. Wilk's Lambda test was used to assess the statistical significance between the groups.

Since no previous study investigating the effect of ECP on the changes of the above biomarkers are available, the study was deemed as a pilot study. We decided to have at least 25 sample sizes for each arm. This sample size determination was calculated to have a power of 90% and with an expected small standardized difference of 0.1–0.3 (23). All hypothesis tests were two-sided with a significance level set at P < 0.05.

### Ethical Clearance

This study had been reviewed and received ethical approval by the Research Ethics Committee/Independent Review Board (REC/IRB) of National Cardiovascular Center—Harapan Kita Hospital, Jakarta—Indonesia, No: 02.01/VII/226/KEP 059/2017, on Dec 21, 2017. All participants have provided their written consent.

## Results

### Patient Characteristics

**Figure 1:**
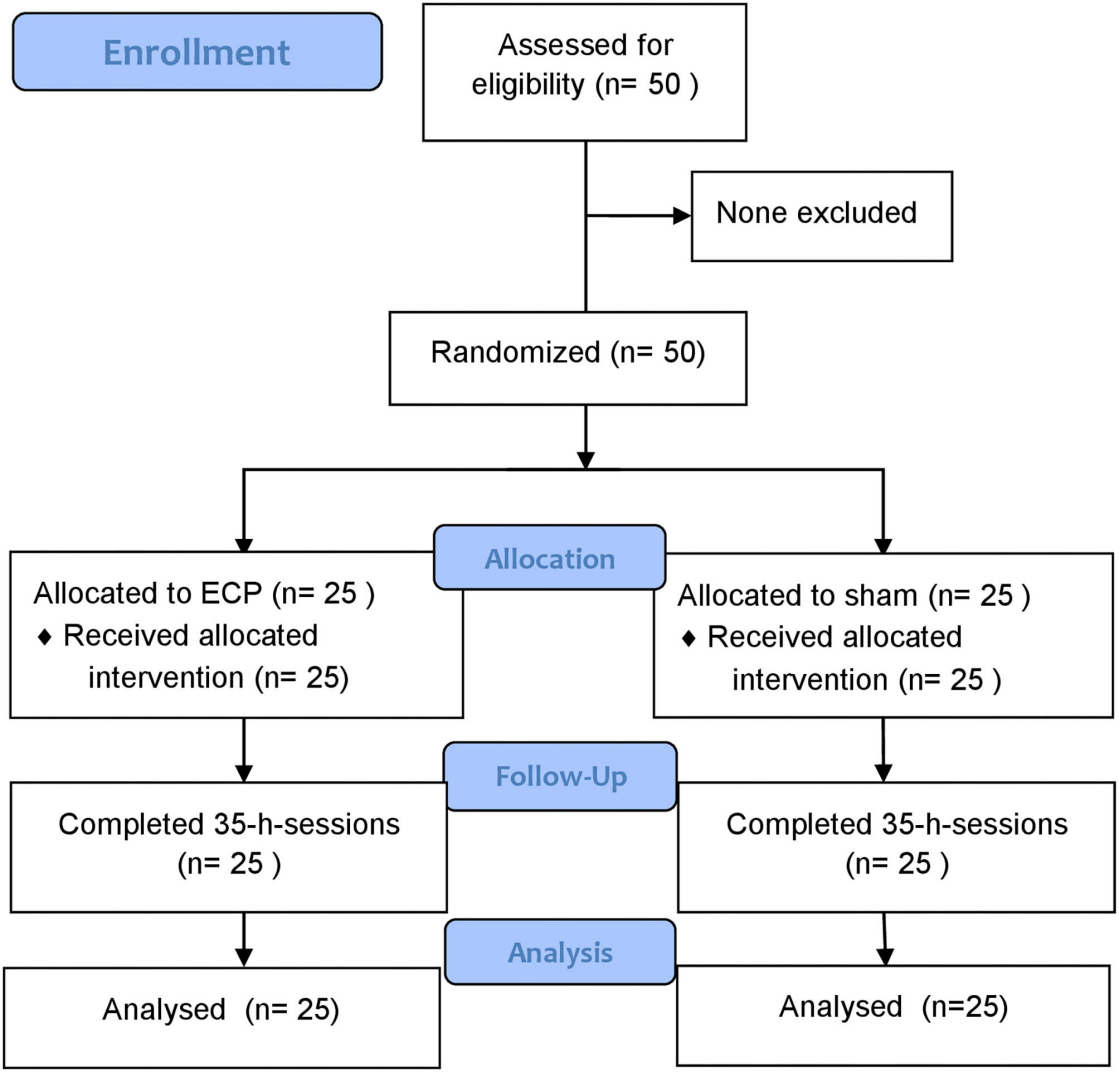
CONSORT flow diagram.

Our study population consisted of 50 patients with RA who have been documented as having CAD, 25 patients in the ECP group, and 25 patients in the sham group. All patients completed the full course of treatment and blood sampling and were included in the final analysis (Figure 1). The baseline characteristics of these patients are shown in Table 1. Effective hemodynamic augmentation was reached in the ECP group with a mean diastolic-to-systolic ratio of 1.2 compared to only 0.8 in the sham group (Table 1).

**Table 1:** Baseline characteristics.

**Variables**	**ECP (*n* = 25)**	**Sham (*n* = 25)**	***P* value**
Age, n (%)			0.57
≥60 years	11 (44)	13 (52)	
<60 years	14 (56)	12 (48)	
^a^Gender, *n* (%)			
Male	23 (92)	21 (84)	0.67
Female	2 (8)	4 (16)	
SBP (mmHg)	124.1 ± 9.4	117.1 ± 7.5	0.19
DBP (mmHg)	67.1 ± 3.4	66.1 ± 0.3	0.70
BMI (kg/m^2^)	26.7(22 to 31.8)	24(21 to 29)	0.02
LVEF (%)	52.1 ± 6	49.1 ± 8	0.52
^a^Previous CABG, *n* (%)	1 (4)	7 (28)	0.05
Previous PCI, *n* (%)	15 (60)	9 (36)	0.09
Diabetes mellitus, *n* (%)	15 (60)	11 (44)	0.26
Smoking, *n* (%)	19 (76)	17 (68)	0.53
Alcohol, *n* (%)	8 (32)	6 (24)	0.53
Hypertension, *n* (%)	15 (60)	10 (40)	0.16
Dyslipidemia, (%)	19 (76)	14 (56)	0.14
Stroke, *n* (%)	6 (24)	5 (20)	0.73
Familial history of CAD, *n* (%)	14 (56)	10 (40)	0.26
Physically active, *n* (%)	19 (76)	15 (60)	0.23
^a^Aspirin, *n* (%)	22 (88)	19 (76)	0.46
Clopidogrel, *n* (%)	16 (64)	18 (72)	0.54
^a^Ticagrelor, *n* (%)	3 (12)	1 (4)	0.61
^a^Nitrat, *n* (%)	20 (80)	23 (92)	0.42
ACE inhibitor, *n* (%)	7 (28)	6 (24)	0.75
ARB, *n* (%)	13 (52)	16 (64)	0.39
^a^Beta blocker, *n* (%)	24 (96)	22 (88)	0.61
^a^Statin, *n* (%)	25 (100)	22 (88)	0.24
^a^Trimetazidine, *n* (%)	4 (16)	4 (16)	1.00
Diuretic, *n* (%)	16 (64)	14 (56)	0.56
CCB, *n* (%)	14 (56)	8 (32)	0.09
6MWT (m)	341.2 (±80)	322.8 (±85)	0.42
CCS class	2 (1 to 4)	2 (1 to 3)	0.25
QoL WHO-5 Well-Being Index	68.5 (±14.5)	68.2 (±14)	0.95
Diastolic/Systolic Ratio	1.2(±0.25)	0.8 (±0.15)	<0.0001

*n, absolute number; ECP, external counterpulsation; SBP, systolic blood pressure; DBP, diastolic blood pressure; BMI, body mass index; LVEF, left ventricle ejection fraction); CABG, coronary artery bypass graft; PCI, percutaneous coronary intervention; CAD, coronary artery disease; ACE, angiotensin converting enzyme; ARB, angiotensin receptor blocker; CCB, calcium channel blocker; 6MWT, six-min walking test; CCS, Canadian Cardiovascular Scoring; NYHA, New York Heart Association; QoL WHO-5, Quality of Life measured by WHO-5 Well-Being Index*.

a *Analysis by Fisher's test (do not qualify for chi-square test)*.

*Normally distributed value is presented as mean (± SD) if not normally distributed is presented as median (mininum–maximum)*.

*Normality test by Shapiro–Wilk, homogeneity test by Levene's test*.

*Numeric variables were analyzed by independent-t-test or Mann–Whitney test*.

All patients assigned to the ECP group completed their 35-hour sessions, so did the sham group. There were no significant differences in the baseline characteristics between the groups, except for body mass index (BMI); male sex was predominantly observed than those of the female sex. The patients in the ECP group were significantly more overweight [26.7 (22–31.8) vs. 24 (21–29), *P* = 0.02] than those in the sham group. The cardiovascular risk factors were comparable between the groups. Diabetes mellitus, hypertension, dyslipidemia, and family history of CAD proportions were more than half encountered in patients. About one-fifth of them have suffered from a stroke. All patients in both groups similarly received the optimal treatment for chronic coronary syndrome, as presented in Table 1. No major adverse cardiovascular events were observed during the study period.

### ECP Preserved VEGF-A and VEGFR-2 Levels

**Figure 2:**
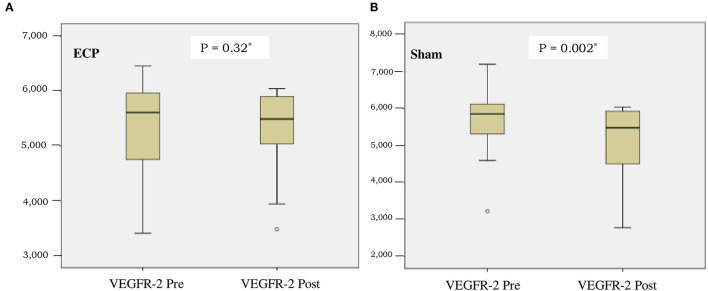
Boxplot of vascular endothelial growth factor receptor-2 (VEGFR-2) concentration before and after intervention in external counter pulsation (ECP) group **(A)** and sham group **(B)**. *Analysis by Wilcoxon test.

The level of VEGF-A in the ECP group was significantly preserved compared to the sham group [ΔVEGF-A 1 pg/ml (−139 to +160) vs. −136 pg/ml (−237 to +67); consecutively, *P* = 0.026] (Table 2). As shown in Figure 2, VEGFR-2 level was also preserved in the ECP group (Figure 2A) but decreased significantly in the sham group (Figure 2B). The reduction of VEGFR-2 was larger in the sham group compared to the ECP group [-517 pg/ml (−1,549 to +1,407) vs.−171 (−844 to +1,166); consecutively, *P* = 0.021]. A negative mark denoted that the levels of VEGF-A and VEGFR-2 decreased in the post-ECP and sham procedures (Table 2; Figure 2).

**Table 2:** ΔVEGF-A and ΔVEGFR-2 after-before intervention in ECP and sham group.

**^a^,^b^Variables**	**ECP (*n* = 25)**	**Sham (*n* = 25)**	***P-*value**
ΔVEGF-A level (pg/ml)	1 (−139 to160)	−136 (−237 to 67)	0.026
ΔVEGFR-2 level (pg/ml)	−171 (−844 to +1,166)	−517 (−1,549 to +1,407)	0.021

*Δ, delta or change; n, absolute number; ECP, external counter pulsation; VEGF-A, vascular endothelial growth factor-A; VEGFR-2, vascular endothelial growth factor receptor-2; pg/ml, picogram/milliliter*.

a *Normality test by Shapiro–Wilk*.

b *Analysis by Mann–Whitney test*.

### Levels of miR-92 Increased After the Procedures

**Figure 3:**
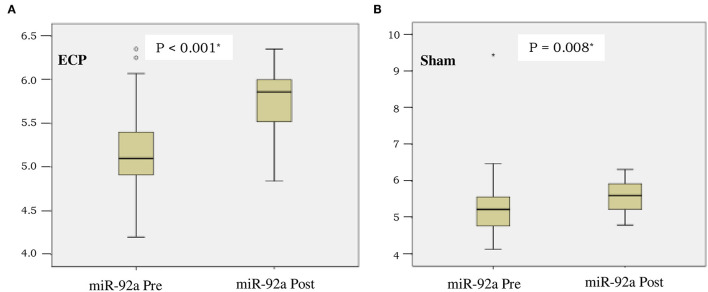
Boxplot of microRNA-92a (miR-92A) expression before and after intervention in ECP group **(A)** and sham group **(B)**. *Analysis by Wilcoxon test.

The concentration of miR-92a was comparable between the groups before the procedures were conducted (*P* = 0.68). After completion of the procedures, the miR-92a level in plasma increased significantly in ECP [as shown in Figure 3A, from 5.1 (4.2–6.4) to 5.9 (4.8–6.4), *p* < 0.001] and sham group (as shown in Figure 3B, from 5.2 (4.1–9.4) to 5.6 (4.8–6.3), *p* = 0.008]. Delta changes and *fold changes* tended to be larger in ECP group although not reaching statistically significant differences in sham group [delta ECP 0.7(−0.5 to +1.6) vs. delta sham 0.5 (−4.2 to +1.2), *p* = 0.33; *fold changes* ECP = 4.6 (0.3–36.5) vs. sham 2.8 (0–15), *p* = 0.33)] (Table 3).

**Table 3:** MicroRNA-92a expression in ECP group and sham group, before and after intervention.

**Variables**	**ECP (*n* = 25)**	**Sham (*n* = 25)**	***P* value^a^**
^*^miR-92a expression, before	5.1 (+4.2 to +6.4)	5.2 (+4.1 to +9.4)	0.68
^*^miR-92a expression, after	5.9 (+4.8 to +6.4)	5.6 (+4.8 to +6.3)	0.09
Delta mir-92a, after-before	0.7 (-0.5 to +1.6)	0.5 (−4.2 to +1.2)	0.33
*^+^Fold-changes* mir-92a, after: before	4.6 (0.3 to 36.5)	2.8 (0 to 15)	0.33

*n, absolute number; ECP, external counter pulsatio); miR-92a, microRNA-92a*.

a *Analysis by Mann–Whitney test, presented as median (min-max)*.

* *miR-92a expression was the result of formula 2^−Δct^ transformed by multiplying with 10^6^ then log 10*.

+ *Fold changes of miR-92a expression were the result of formula 2^−ΔΔct^*.

### Effect of ECP on Dependent Variables

Although there was an imbalance of BMI between the groups, the effect of ECP on angiogenesis remained significant after adjustment for BMI (Table 4). MANCOVA test was used to distinguish the two groups with multiple dependent variables.

**Table 4:** Multivariate analysis the effect of ECP on dependent variables.

**Risk Factors**	**Delta VEGF-A**			**Delta VEGFR-2**			**Delta miR-92a**		
	**Partial Eta Square**	**B coefficient**	***P*-value^a^**	**Partial Eta Square**	**B coefficient**	***P*-value^**a**^**	**Partial Eta Square**	**B coefficient**	***P*-value^a^**
BMI	0.006	2.853	0.597	0.005	−17.223	0.636	0.036	0.042	0.195
ECP	0.083	56.664	0.047	0.094	409.625	0.034	0.006	0.089	0.593

*ECP, external counter pulsation; VEGF-A, vascular endothelial growth factor-A; VEGFR-2, vascular endothelial growth factor receptor-2, miR-92a: microRNA-92a*.

a *Analysis by MANCOVA and Wilk–Lambda test*.

### ECP and Clinical Effect

Although there were no significant differences observed between the groups, the ECP group tended to show an improved aerobic capacity detected by 6MWT, QoL measured by the WHO-5 Well Being Index, and ejection fraction (EF) (Table 5).

**Table 5:** Differences in CCS class, 6MWT and QoL WHO-5 in ECP and sham group, before and after intervention.

**Variable**	**ECP (*n* = 25)**	**Sham (*n* = 25)**	***P* Value**
^a^, ^b^Δ CCS Class	−1 (−2 to 0)	−1 (−2 to −1)	NS
^a^, ^b^Δ 6MWT (m)	41 (−58 to +330)	19 (−55 to +190)	NS
^a^, ^b^Δ QoL WHO-5	8 (0 to +32)	4 (−6 to +28)	NS
^a^, ^c^Δ EF (%)	1.3 (±14)	−0.32 (±11)	NS

*Δ, delta or change; ECP, external counter pulsation; CCS, Canadian Cardiovascular Society; 6MWT, 6-min walk test; QoL, quality of life; WHO, World Health Organization*.

a *Normality test by Shapiro-Wilk test*.

b *Mean difference analysis was performed by Mann-Whitney test*.

c *Mean difference analysis was performed by Independent-t-test*.

* *NS: Not Significant*.

## Discussion

There is a growing prevalence of refractory ischemia due to residual CAD even after revascularization procedures, such as PCI and/or CABG. As a matter of fact, as shown in Table 1, 16 of the 25 patients in each group have had previous revascularizations with PCI + CABG. Thus, 64% of the patients with RA in each group had residual CAD with recurrent ischemia.

This study has demonstrated that in patients with RA, ECP maintains angiogenesis, as measured by the data showing that VEGF-A level in the ECP group remains stable. On the contrary, VEGF-A significantly decreases in the sham group. Similarly, the significant preservation of angiogenesis in the ECP group is also demonstrated by a more significant reduction of VEGFR-2 in the sham group than in the ECP group (Table 2; Figure 2). An imbalance of BMI between the groups might be due to chance. Furthermore, after being adjusted using MANCOVA analysis, the ECP still reveals a significant effect on the changes in angiogenesis (Table 4). This evidence might be pointed out by the data proved by Ebos et al. (24), which confirmed that VEGF-A binding to and activation of VEGFR-2 leads to downregulation and a decrease in VEGFR-2 production in angiogenesis. A previous study also demonstrated that increased VEGF-A production might induce angiogenesis by initiating reactive oxygen species-endoplasmic reticulum (ER) stress-autophagy axis in endothelial cells (25).

The acute effect of the ECP is to increase the coronary blood flow and improve endothelial function (26) during diastole while reducing the cardiac workload at the onset of systole. Although the long-term mechanism is yet unknown, it is thought to result from an increase in endothelial shear stress (ESS), wherein it stimulates collateralization and angiogenesis to the ischemic region, among other mechanisms (27, 28). The high-shear stress of ECP, which comprises radial, circumferential, and longitudinal forces, will inhibit atherosclerosis (29, 30). Increased ESS would increase nitric oxide (NO) production (31) and VEGF-A (32).

The complex process of mechano-reception and mechano-transduction, leading to pro-atherosclerosis or anti-atherosclerosis state, is regulated by micro-RNA (miR). The miR is a small noncoding RNA that post-transcriptionally controls gene expression and regulates a wide range of physiological and pathophysiological processes (33). Among them, miR-92a has been shown as one valuable therapeutic target in the setting of ischemic disease. The miR-92a was highly expressed in human endothelial cells, and it controlled the growth of new angiogenesis. Forced overexpression of miR-92a in human endothelial cells blocked sprout formation in angiogenesis, inhibited vascular network formation, and reduced endothelial cell migration (34). Meanwhile, inhibition of miR-92a increased angiogenesis in mice after ischemia and also enhances ischemic tissue repair, improves endothelial dysfunction, reduces inflammation, and stabilizes atherosclerotic plaques (34–36).

Previous studies have shown that miR-92a is a flow-sensitive miRNA, whose expression was increased in low ESS exposure, while decreased in a high laminar or pulsatile ESS (37). In contrast, our study showed that miR-92a was increased in patients with RA after ECP intervention. However, it should be pointed out that in the aforementioned studies, the reduction of miR-92a was observed from *in vitro* studies conducted in a 24-hour continuous exposure of laminar and a high-ESS in cell culture medium, then intracellular miR-92a was measured (36, 38). Meanwhile, our study measured circulating miR-92a in plasma. Whether the circulating level of miRNA could affect intracellular gene expression or if the circulating miRNA level correlates with the intracellular level needs to be confirmed. The increase in circulating miR-92a level might indicate the release from cell and significantly decreased the uptake by target cell, which might mitigate its intracellular proatherosclerotic level (39). Alternatively, other miRNAs might have played a more important role in this regulation.

Whether the increase in circulating miR-92a is a favorable regulation or a counterregulatory effect is still unknown. However, a study by Marfella et al. (20) has shown that the level of miR-92a in circulation increased in patients with heart failure who experienced an improvement in EF and left ventricle dimension after cardiac resynchronization therapy (CRT). Studies in cancer tissues have also shown that miR-92a regulates PTEN/AKT signaling pathway by inhibiting PTEN, thus, activates AKT signaling (40, 41). Activation of AKT signaling results in cell cycle progression, survival, metabolism, and migration. The AKT pathway also plays an important role in angiogenesis stimulation through the effect on endothelial cell and other cells that produce angiogenesis signals, such as tumor cells (42).

Our subjects were all patients with RA having severe long-standing CAD and endothelial dysfunction. Thus, the ischemic myocardium and dysfunctional endothelial cell may produce distinct responses compared to normal endothelial cell in the culture medium. Accordingly, a study by Song et al. (43) showed that cardiomyocyte exposed to 48 h of hypoxia expressed higher miR-92a compared to those in normoxia condition. Thus, a comparison with a normal subject might be needed to evaluate the response to ECP. Alternatively, increased expression of miR-92a is possibly due to homeostasis counter-regulatory phenomenon in the ischemic myocardium of patients with RA as a result of enhanced angiogenesis in the ECP group by high-ESS. The previous study has shown that vascular endothelial growth factor (VEGF) overexpression could increase vascular permeability and tissue oedema, pericardial effusion, and angioma formation (44).

Interestingly, circulating miR-92a also significantly increases in the sham group so that the difference between the two groups becomes not significant. Generally, *in vitro* studies of miR-92a utilizes a perfusion system to generate shear stress of about 12 dynes/ cm^2^ (16, 36). This value is lower than the shear stress generated by ECP in the human and animal study, which ranges from 20 to 40 dynes/cm^2^ (29, 45). Thus, sham treatment with 75 mmHg pressure given might also result in increased ***shear*** ***stress*** to some level that might also regulate miR-92a.

Although not statistically significant, ECP tended to increase the QoL and aerobic capacity compared to sham. However, this study was underpowered to evaluate these clinical endpoints. The previous randomized sham-controlled trial has shown that ECP significantly reduced anginal frequency and increased exercise-induced ischemia time (46). A registry-based study also showed an improvement in angina class and frequency, as well as an improvement in the QoL, which persisted up until 3 years after therapy in patients with or without heart failure (5, 47). A meta-analysis by Zhang et al. (48) which included 18 studies with a total of 1,768 patients confirmed that ECP resulted in the improvement of at least one angina class in 85% of patients. Biomarker studies have shown an increase in NO synthesis and a decrease in endothelin-1 following ECP indicating an improvement in endothelial function (31, 49) which is in accordance with our study that shows better angiogenesis marker after ECP treatment. However, further studies are needed to evaluate whether improvement in angiogenesis marker will be sustained in the long term after completion of ECP and its relation to the clinical outcome. A sub-analysis study from Pravian et al. (50) has shown that although ECP did not improve left ventricular (LV) longitudinal strain globally or segmentally, there was an improvement in segments with post-systolic shortening (PSS) which indicates improvement in myocardial perfusion.

No serious adverse effect occurred during the treatment in both groups. However, we acknowledge that there are some limitations to the study. First, there was an imbalance of BMI variables between the groups, although it does not affect the conclusion. Second, ECP has the drawbacks of its high cost and is not covered by our national health insurance, thus limiting its availability and accessibility. Its cumbersome technique also requires a specialized technician and long-term commitment from the patient to complete the whole treatment session. Third, we did not include normal healthy subjects to compare the response to shear stress by ECP on VEGF-A, VEGFR-2, or miR-92a level because of ethical considerations and our hospital standard care restriction. Fourth, we did not provide a comparison of circulating miR-92a with its intra-cellular expression. Fifth, we only used the ELISA method to measure the levels of VEGF-A and VEGFR-2 protein in plasma. Future studies incorporating several measurement techniques are needed to achieve a more solid conclusion. Furthermore, larger studies are needed to ascertain the benefit of ECP on angiogenesis marker, miR-92a, CCS, 6MWT, and QoL. Interestingly, several other invasive options to reduce angina symptoms in patients with RA, such as cell-based therapies, gene therapy, spinal cord stimulation (SCS), trans-myocardial laser revascularization (TMLR), and coronary sinus reduction can also serve as a promising option and necessitate further investigation (4, 17).

## Conclusions

In summary, the present study demonstrates that ECP may improve angiogenesis by preserving the expression of VEGF-A and VEGFR-2. However, both ECP and sham increase miR-92a circulating level significantly, and the number of changes was not different between the two groups.

## Data Availability Statement

The original contributions presented in the study are included in the article/Supplementary Material, further inquiries can be directed to the corresponding author/s.

## Ethics Statement

The studies involving human participants were reviewed and approved by Research Ethics Committee/Independent Review Board (REC/IRB) of National Cardiovascular Center – Harapan Kita Hospital, Jakarta – Indonesia. The patients/participants provided their written informed consent to participate in this study.

## Author Contributions

AA, AS, GL, EZ, BD, RS, BR, SH, SA, DH, DZ, EEl, and AM contributed to the conception and design of the study. GL and EZ organized the database. EEk and SW designed, performed laboratory examinations, and analyzed the result. GL and EZ performed the statistical analysis. AA, AS, EZ, and GL wrote the first draft of the manuscript. BD, RS, BR, SH, EEk, SW, SA, DH, DZ, EEl, and AM wrote sections of the manuscript. All authors contributed to manuscript revision and read and approved the submitted version.

## Funding

This study was funded by the National Health Research and Development of the Ministry of Health of Indonesia (No: HK.02.02/I/27/2020). Support for the laboratory and data analysis was provided by the National Cardiovascular Center, Harapan Kita Hospital, Jakarta. Open access publication fees payable by authors will be partially reimbursed by a Grant from the National Health Research and Development of the Ministry of Health of Indonesia as part of the agreement.

## Conflict of Interest

The authors declare that the research was conducted in the absence of any commercial or financial relationships that could be construed as a potential conflict of interest.

## Publisher's Note

All claims expressed in this article are solely those of the authors and do not necessarily represent those of their affiliated organizations, or those of the publisher, the editors and the reviewers. Any product that may be evaluated in this article, or claim that may be made by its manufacturer, is not guaranteed or endorsed by the publisher.
